# Evaluation of Three Automated Extraction Systems for the Detection of SARS-CoV-2 from Clinical Respiratory Specimens

**DOI:** 10.3390/life12010068

**Published:** 2022-01-04

**Authors:** Ho-Jae Lim, Hye-Soo Jung, Min-Young Park, Young-Hyun Baek, Balaji Kannappan, Jin-Young Park, Jae-Hyun Yang, Ja-Hwan Seol, Min-Woo Lee, Sun-Kyung Jung, Sun-Hwa Lee, Jung-Eun Park, Yong-Jin Yang

**Affiliations:** 1 Department of Molecular Diagnostics, Seegene Medical Foundation, Seoul 04805, Korea; 52rotc.hjl@mf.seegene.com (H.-J.L.); hsjung@mf.seegene.com (H.-S.J.); pyli186@mf.seegene.com (M.-Y.P.); baek0h@mf.seegene.com (Y.-H.B.); skjung@mf.seegene.com (S.-K.J.); lshkim@mf.seegene.com (S.-H.L.); 2 BK21 FOUR Educational Research Group for Age-Associated Disorder Control Technology, Department of Integrative Biological Sciences, Chosun University, Gwangju 61452, Korea; balajikannappan@chosun.ac.kr; 3 Department of Neuroscience, Mayo Clinic, Jacksonville, FL 32224, USA; Park.Jinyoung@mayo.edu; 4 Paul F. Glenn Center for Biology of Aging Research, Department of Genetics, Blavatnik Institute, Harvard Medical School, Boston, MA 02115, USA; Jae-Hyun_Yang@hms.harvard.edu; 5 Department of Molecular Medicine, Institute of Biotechnology, University of Texas Health Science Center at San Antonio, 7703 Floyd Curl Drive, San Antonio, TX 78229, USA; SeolJ@uthscsa.edu; 6 Department of Integrated Biomedical Science, Soonchunhyang Institute of Medi-Bio Science (SIMS), Soonchunhyang University, Cheonan-si 31151, Korea; mwlee12@sch.ac.kr

**Keywords:** SARS-CoV-2, mRT-qPCR, automated extraction system

## Abstract

Severe acute respiratory syndrome coronavirus (SARS-CoV-2) is highly contagious and causes coronavirus disease 2019 (COVID-19). Reverse transcription quantitative polymerase chain reaction (RT-qPCR) is the most accurate and reliable molecular assay to detect active SARS-CoV-2 infection. However, a rapid increase in test subjects has created a global bottleneck in testing capacity. Given that efficient nucleic acid extraction greatly affects reliable and accurate testing results, we compared three extraction platforms: MagNA Pure 96 DNA and Viral NA Small Volume kit on MagNA Pure 96 (Roche, Basel, Switzerland), careGENE^TM^ Viral/Pathogen HiFi Nucleic Acid Isolation kit (WELLS BIO Inc., Seoul, Korea) on KingFisher Flex (Thermo Fisher Scientific, Rocklin, CA, USA), and SGRespi^TM^ Pure kit (Seegene Inc., Seoul, Korea) on Maelstrom 9600 (Taiwan Advanced Nanotech Inc., Taoyuan, Taiwan). RNA was extracted from 245 residual respiratory specimens from the different types of samples (i.e., NPS, sputum, and saliva) using three different kits. The 95% limits of detection of median tissue culture infectious dose per milliliter (TCID_50_/mL) for the MagNA Pure 96, KingFisher Flex, and Maelstrom 9600 were 0.37–3.15 × 10^1^, 0.41–3.62 × 10^1^, and 0.33–1.98 × 10^1^, respectively. The KingFisher Flex platform exhibited 99.2% sensitivity and 100% specificity, whereas Maelstrom 9600 exhibited 98.3–100% sensitivity and 100% specificity. Bland–Altman analysis revealed a 95.2% concordance between MagNA Pure 96 and KingFisher Flex and 95.4% concordance between MagNA Pure 96 and Maelstrom 9600, indicating that all three platforms provided statistically reliable results. This suggests that two modifying platforms, KingFisher Flex and Maelstrom 9600, are accurate and scalable extraction platforms for large-scale SARS-CoV-2 clinical detection and could help the management of COVID-19 patients.

## 1. Introduction

Severe acute respiratory syndrome coronavirus 2 (SARS-CoV-2) is the causative agent of coronavirus disease 2019 (COVID-19). The COVID-19 outbreak began in December 2019 [1] and was declared a global pandemic on 11 March 2020 by the World Health Organization (WHO) [2]. As of 21 December 2021, more than 274 million confirmed cases and 5.3 million deaths due to COVID-19 had been reported [3]. SARS-CoV-2 is predominantly transmitted by exposure to respiratory droplets and aerosol particles [4]. As SARS-CoV-2 is easily transmitted through the air and has a relatively long incubation time, several types and grades of measures, including travel restrictions, social distancing, and limitations on movement, have been adopted by the governments to prevent the spread of this virus [5,6,7].

The United States Centers for Disease Control and Prevention (CDC) has established a decision tree for four indications and a standard assay for COVID-19 testing. Briefly, these include individuals with symptoms of COVID-19, those who have been in close contact with a person confirmed as having COVID-19, unvaccinated people who have attended a large social gathering or been in poorly ventilated indoor settings, and subjects referred for testing by the associated health department [8]. The CDC recommends a standard assay that includes RNA extraction from a nasopharyngeal swab (NPS), followed by a reverse transcription quantitative polymerase chain reaction (RT-qPCR) to detect purified SARS-CoV-2 RNA [9].

The CDC recommended a list of automated technology commercial extraction kits (Roche, QIAGEN, and bioMérieux) for sample preparation upstream of the emergency use authorization COVID-19 RT-PCR diagnostic test [10]. One of the QIAGEN kits, QIAamp DSP Viral RNA (QIAGEN, Hilden, Germany), involves column-based purification, whereas the other QIAGEN kits, such as the EZ1 DSP Virus kit, involve magnetic particle-based purification, as do the Roche kits, including the Total Nucleic Acid, Nucleic Acid Isolation I, and MagNA Pure 96 DNA and Viral NA Small Volume kit (Roche, Basel, Switzerland) [10]. All of these kits have been used for nucleic acid extraction from respiratory tract specimens. However, the high demand for these kits has created a global bottleneck in testing capacity [11]. The modification of resources and methods, including extraction-free methods, may enable us to process a large number of multiplex RT-qPCR samples for detecting SARS-CoV-2 RNA [12]. However, the protocols for extraction-free methods differ among specimen types, as the ability to efficiently detect viral RNA is affected by media concentration and inhibitors, resulting in potentially insufficient sensitivity [13,14].

Thus far, the MagNA Pure96 platform (Roche, Basel, Switzerland) verified by previous studies is an accurate standard protocol [15]. However, careGENE^TM^ Viral/Pathogen HiFi Nucleic Acid Isolation kit (WELLS BIO Inc., Seoul, Korea) on the KingFisher Flex platform (Thermo Fisher Scientific, Rocklin, CA, USA) and the SGRespi^TM^ Pure kit (Seegene Inc., Seoul, Korea) on the Maelstrom 9600 platform (Taiwan Advanced Nanotech Inc., Taoyuan, Taiwan) have been recently developed; it is suggested that these novel extraction systems and platforms may extract nucleic acids as accurately as, but more rapidly than, the existing MagNA Pure96 platform [16,17,18]. Therefore, herein, the efficiency and extraction performance of these new extraction systems and platforms were evaluated and compared with those of the MagNA Pure 96 platform for the detect of SARS-CoV-2 in 245 respiratory specimens (NPS, sputum, and saliva).

## 2. Materials and Methods

### 2.1. Clinical Specimens and Automated Extraction Systems

Anonymized residual respiratory specimens from 245 patients, including 82 NPS, 79 sputum, and 84 saliva samples, were obtained from February to June 2021 as part of the routine procedure for SARS-CoV-2 testing. These archived samples included 57 NPS, 45 sputum, and 18 saliva samples positive for SARS-CoV-2, and 25 NPS, 34 sputum, and 66 saliva samples negative for the virus. All procedures were approved by the institutional review boards at the Seegene Medical Foundation (SMF-IRB-2021-005). Sputum and saliva specimens were each completely homogenized in 3 mL of 1x phosphate-buffered saline (PBS, pH 7.2). Three 200 μL aliquots were obtained from each sample, including NPS samples, for simultaneous nucleic acid extraction on three platforms: the MagNA Pure 96 DNA and Viral NA Small Volume kit (Roche, Basel, Switzerland) on the MagNA Pure 96 platform (Roche, Basel, Switzerland), the careGENE^TM^ Viral/Pathogen HiFi Nucleic Acid Isolation kit (WELLS BIO Inc., Seoul, Korea) on the KingFisher Flex platform (Thermo Fisher Scientific, Rocklin, CA, USA), and the SGRespi^TM^ Pure kit (Seegene Inc., Seoul, Korea) on the Maelstrom 9600 platform (Taiwan Advanced Nanotech Inc., Taoyuan, Taiwan). The main characteristics of these platforms that are compared in this study, such as the platform manufacturer, properties, measurement, extraction reagent, number of samples, extraction technique, duration of extraction, sample loading, nucleic acid collection, and identification reader, are summarized in Table 1.

### 2.2. Total Nucleic Acids Extraction

Nucleic acids were simultaneously extracted from the samples using the automated MagNA Pure 96 platform, KingFisher Flex platform, and Maelstrom 9600 platform. For extraction using the MagNA Pure 96 platform, the MagNA Pure 96 DNA and Viral NA Small Volume kit was used and the extraction was performed following the manufacturers’ instructions [19]. Briefly, 200 µL of sample was transferred to the processing cartridge, which was loaded on the instrument along with two additional empty cartridges, two reagent trays, pipette tips on holders, elution plates, and glass magnetic particles. Following the “Pathogen Universal 200” protocol, the sample was mixed with 250 µL lysis/binding buffer and magnetic particles. Subsequently, the nucleic acids bound to the beads were separated using a magnetic separator and washed twice to remove any residual contaminants. Pure nucleic acids were then eluted in 100 µL of elution buffer.

For extraction using the KingFisher Flex platform, the careGENE^TM^ Viral/Pathogen HiFi Nucleic Acid Isolation kit was used as per the manufacturers’ “MVP Flex” protocol. Briefly, 200 µL of sample was transferred to 300 µL of lysis buffer cartridge containing glass magnetic particles, which was loaded on the instrument along with three washing reagent cartridges, pipette tips on holders, and elution plates. Extraction was performed under the following conditions: 70 °C for 9 min (lysis/binding), followed by washing of cartridge 1 for 3 min, cartridge 2 for 2 min, and cartridge 3 for 0.5 min. After washing thrice, the nucleic acid bound magnetic beads were dried for 2 min. Pure nucleic acid was eluted at 70 °C in 80 µL of elution buffer for 6 min.

For extraction using the Maelstrom 9600 platform, the SGRespi^TM^ Pure kit (Seegene Inc., Seoul, Korea) was used following the manufacturer’s “SG-RESPI” protocol. Briefly, 200 µL of sample was transferred to the lysis buffer cartridge, which was loaded on the instrument along with three washing reagent cartridges, pipette tips on holders, and elution plates. The third washing cartridge contained the glass magnetic particles. Extraction was performed under the following conditions: the glass magnetic particles were transferred from the washing buffer cartridge 3 to the lysis buffer cartridge for 1 min, 94 °C for 4 min (lysis/binding), followed by an initial washing for 0.3 min and a second washing for 0.6 min. After washing twice, the nucleic acid bound magnetic beads were dried for 4 min and then eluted at 80 °C in 100 µL of elution buffer for 5 min. After successful extraction, the elution plate was sealed and removed for PCR usage. A maximum of 96 specimens can be processed by the three platforms in each run.

### 2.3. Multiplex RT-qPCR Analysis

The multiplex RT-qPCR (mRT-qPCR) was performed using Allplex™ SARS-CoV-2 Assay kits (Seegene Inc., Seoul, Korea) to detect SARS-CoV-2 RNA following the manufacturers’ instructions under the following cycling conditions: 50 °C for 20 min (reverse transcription), followed by 95 °C for 15 min (initial denaturation), and 45 cycles of 95 °C for 10 s (denaturation), 60 °C for 15 s (annealing), and 72 °C for 10 s (extension) [20,21]. A sample was considered positive if more than one cycle threshold (Ct) value was under 36, regardless of the results of the internal control [22].

### 2.4. Efficiency of the Extraction Systems

The efficiencies of the three extraction systems were compared using the commercially available heat-inactivated viral culture fluids of the SARS-CoV-2 isolate 0810587CFHI obtained from Zeptometrix (Buffalo, NY, USA). Five replicates with four different concentrations of the viral isolate were used for this assessment. The viral culture was serially diluted (10^−4^–10^−7^) using Gene Transport Medium (GeneTM; SG Medical Inc., Seoul, Korea). RNA was extracted from the serially diluted viral stock using the three extraction systems. mRT-qPCR assay was performed using Allplex™ SARS-CoV-2 Assay kit following the manufacturer’s protocol. Ct values above 36 were not considered for evaluating extraction efficiency.

### 2.5. Limits of Detection (LOD) of the Extraction Systems

The analytical sensitivities of the three extraction systems were compared using two SARS-CoV-2 strains, 0810589CFHI and 0810590CFHI, both obtained from Zeptometrix (Buffalo, NY, USA). These strains were used as median tissue culture infectious doses per milliliter (TCID_50_/mL), as provided by the supplier. The strains were serially diluted (10^−1^–10^−9^) using GeneTM (SG Medical Inc., Seoul, Korea). RNA was simultaneously extracted from 13 mL of serially diluted viral stocks of each strain using the three extraction systems. mRT-qPCR assay was performed using Allplex™ SARS-CoV-2 Assay kits. Ct values above 36 were not considered for evaluating analytical sensitivity.

### 2.6. Statistical Analysis

Statistical analyses were performed using SPSS version 26.0 (IBM Corp., Armonk, NY, USA) for Mac and R-studio (Rstudio, Boston, MA, USA) for Windows. The coefficient of variation (CV) was determined to evaluate the extraction performance using measurements obtained from triplicate runs and presented as means and standard deviations; CV values < 4% were acceptable [23]. The extraction efficiency was calculated from the slope of the linear regression using the following formula: E value = 100 × (−1 + 10^−1/slope^) [24]. The LOD of mRT-qPCR assays and the concentrations of the sample detected as positive with 95% confidence were estimated to fit the probit regression model [25]. The sensitivity and specificity of each diagnostic test for SARS-CoV-2 using each system were compared with those of the MagNA Pure 96 platform. The Bland–Altman analysis was performed using all three platforms by positive matching for each variable. The presence of proportional bias was determined by testing the slope of the regression line fitted to the Bland–Altman plot [26].

## 3. Results

### 3.1. Comparison of the Three Nucleic Acid Extraction Systems

The three extraction systems exhibited similar characteristics (Table 1), including the sample loading requirements, use of magnetic beads, maximum processing ability of a single cartridge, and system automation. By contrast, when compared with the running time of MagNA Pure (60 min), both KingFisher Flex and Maelstrom 9600 had running times of only 30 min. Furthermore, given the machine measurements, the installation space occupied by one MagNA Pure 96 was equivalent to that of six KingFisher Flex instruments or three Maelstrom 9600. Compared with that of KingFisher Flex, the identification method for MagNA Pure 96 involved barcode detection to discriminate the reagent and sample, whereas that for Maelstrom 9600 involved inputting sample information. These differences suggest that both KingFisher and Maelstrom 9600 systems allow using modified reagents as open systems and for a more rapid extraction of nucleic acids in a relatively limited amount of space.

### 3.2. Extraction Performance of the Three Systems

Ct values of SARS-CoV-2 using the MagNA Pure 96, KingFisher Flex, and Maelstrom 9600 platforms were analyzed in 245 samples. Among these, 120 samples were determined as SARS-CoV-2-positive, based on the Ct values. The inter-assay CVs using these three platforms were 1.58–3.28%, 1.32–3.81%, and 1.18–2.48%, for NPS samples; 2.53–3.21%, 1.78–2.16%, and 1.92–2.21%, for sputum samples; and 2.52–3.87%, 1.45–1.76%, and 1.10–1.62%, for saliva samples. These inter-assay CVs were all <3.7%, indicating that the variability was acceptable (Table 2). The analytical performance of the three systems was compared using the 245 samples. Compared with the KingFisher Flex system, the MagNA Pure 96 system had sensitivities of 98.2–100% for NPS, 100% for sputum, and 94.4–100% for saliva samples. By contrast, when compared to the Maelstrom 9600 system, the MagNA Pure 96 system had sensitivities of 100% for NPS and saliva and 97.8–100% for sputum samples; however, specificity levels were 100% for all samples. These results indicated that the performances of the KingFisher Flex and Maelstrom 9600 systems were comparable with those of the MagNA Pure 96 system (Table 3). Nucleic acid was efficiently extracted by each platform from all types of samples, without inhibition by other constituents of these samples.

### 3.3. Intersystem Comparison of Ct Values

**Figure 1:**
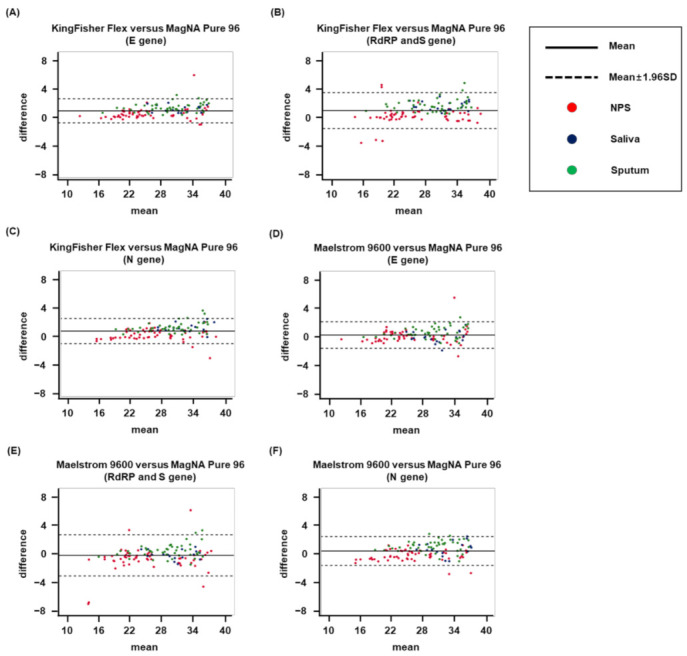
Bland–Altman analysis of the quantitative data from three platforms for SARS-CoV-2. The analyses were performed using matched positive samples from all assays to compare the Ct values from the MagNA Pure 96 and (KingFisher Flex & Maelstrom 9600) platforms. The mean Ct values are plotted on the *x*-axis, and the Ct differences between the two platforms for each sample are plotted on the *y*-axis. The mean and 1.96 SD border are shown. (**A**,**D**) E gene, the gene encoding the envelope protein of SARS-CoV-2; (**B**,**E**) RdRP gene, the gene encoding the RNA-dependent RNA polymerase of SARS-CoV-2 (**B**,**E**); (**C**,**F**) N gene, the gene encoding the nucleocapsid protein of SARS-CoV-2.

A Bland–Altman analysis was performed to assess the correspondence of test results obtained using the MagNA Pure 96 and KingFisher Flex platforms (Figure 1A–C) and the MagNA Pure 96 and Maelstrom 9600 platforms (Figure 1D–F). An analysis of 120 SARS-CoV-2-positive clinical specimens demonstrated an exceptional degree of correspondence between the MagNA Pure 96 and KingFisher Flex platforms, with a 94.1–95.8% consistency in the detection of the E, RdRP, S, and N genes. Similarly, the comparison between the MagNA Pure 96 and Maelstrom 9600 data revealed a 94.2–96.7% consistency in the detection of the same genes. Over 95.2% of samples tested using MagNA Pure 96 and KingFisher Flex, and 95.4% of samples tested using MagNA Pure 96 and Maelstrom 9600, were between the set standard deviation (SD) boundaries, indicating an exceptionally high correlation between the MagNA Pure 96 data and those of the other well-established assays.

### 3.4. Efficiency of the Extraction Systems for SARS-CoV-2

**Figure 2:**
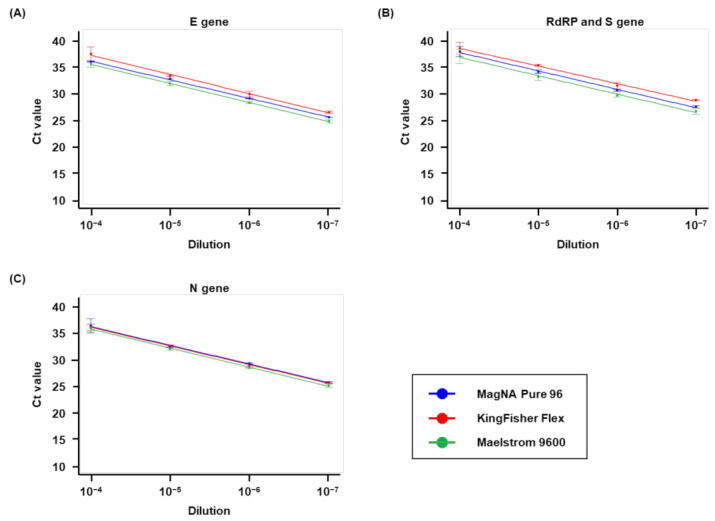
Comparison of the efficiency of three extraction systems. The extraction efficiency of each target gene was assessed using five replicate 10-fold dilution series of SARS-CoV-2 strain. The regression lines are shown. (**A**) E gene, the gene encoding the envelope protein of SARS-CoV-2; (**B**) RdRp and S gene, the gene encoding the RNA-dependent RNA polymerase and the spike protein of SARS-CoV-2; (**C**) N gene, the gene encoding the nucleocapsid protein of SARS-CoV-2; SARS-CoV-2, severe acute respiratory syndrome coronavirus 2.

Extraction efficiencies of the MagNA Pure 96, KingFisher Flex, and Maelstrom 9600 platforms were analyzed (Figure 2). The E value of the MagNA Pure 96 platform was 94–96% for the detection of the E, RdRP, S, and N genes. Similarly, the E values of the KingFisher Flex and Maelstrom 9600 platforms were 89–102% and 91–95%, respectively, for the above-mentioned genes. The R^2^ values for the MagNA Pure 96, KingFisher Flex, and Maelstrom 9600 platforms were 0.975–0.999, 0.974–0.992, and 0.964–0.992, respectively. Therefore, the extraction efficiency of the KingFisher Flex and Maelstrom 9600 platforms was similar to that of the MagNA Pure 96 platform.

### 3.5. Analytical Sensitivity and LOD of the Extraction Systems for SARS-CoV-2

The analytical sensitivity and LOD were estimated with 16 replicates of two positive strains at six different concentrations; the dilutions of a standard solution ranged from 10^−4^ to 10^−9^ (Table 4). The 95% LOD for the Zeptometrix-0810589CFHI strain was approximately 13.4–31.5 TCID_50_/mL for MagNA Pure 96, 9.7–36.2 TCID_50_/mL for KingFisher Flex, and 11.6–19.8 TCID_50_/mL for Maelstrom 9600. However, the 95% LODs for the Zeptometrix-0810590CFHI strain was 0.37–0.53 TCID_50_/mL for MagNA Pure 96, 0.41–0.65 TCID_50_/mL for KingFisher Flex, and 0.33–0.88 TCID_50_/mL for Maelstrom 9600 (Table 4). The assay results showed a 100% reproducibility for all target genes, except for the Maelstrom 9600 N gene, at concentrations as low as 1.4 TCID_50_/mL, but showed similar ranges of LOD thresholds for all three platforms.

## 4. Discussion

The ongoing COVID-19 pandemic requires the testing of increasing numbers of clinical specimens, both to detect infected individuals and prevent the global spread of the virus [27]. The emergence of highly contagious strains has demonstrated that the rapid and highly sensitive detection of SARS-CoV-2 is critical to minimize transmission [28,29]. The high global demand for testing has led to shortages in consumables and reagents required for the extraction and molecular detection of SARS-CoV-2 RNA in samples derived from the respiratory system [11]. Currently, the bottleneck of nucleic acid extraction kits will become more severe due to the spread of the SARS-CoV-2 lineage BA.1 (the Omicron variant). Various approaches, including reagent-free testing and modification of resources, are currently being developed to overcome these shortages and increase the ability to test for SARS-CoV-2 [11,13]. Monitoring the emergence and spread of SARS-CoV-2, using platforms with performances similar to those of the existing platforms, is essential for implementing effective public health strategies [12].

In this study, three extraction platforms were evaluated using the resources of original (MagNA Pure 96) and modified platforms (KingFisher Flex and Maelstrom 9600) from the standard to detect the most frequently examined strains of SARS-CoV-2. All three automated extraction systems (MagNA Pure 96, KingFisher Flex, and Maelstrom 9600) exhibited high extraction efficiencies (Figure 2) and similar LOD for the tested SARS-CoV-2 strains (Table 4). Although the platforms yielded minor differences in LOD, particularly between dilutions of 10^−7^ and 10^−8^, the Poisson distribution and the PCR gray region have shown that the virus in highly diluted samples is typically undetected, as the viral load is very low [30]. These results suggest that the performance of the KingFisher Flex and Maelstrom 9600 extraction platforms are very similar to that of the standardized platform, MagNA Pure 96.

A comparison of the results obtained from the analysis of the clinical samples across different platforms is crucial for determining the effectiveness of nucleic acid extraction and removal of enzymatic inhibitors, which have a direct impact on qPCR results [14,31]. The present study evaluated the clinical reliability and reproducibility of the three above-mentioned platforms. These clinical assessments revealed that the performances of both the KingFisher Flex and Maelstrom 9600 platforms were highly comparable with that of the MagNA Pure 96 platform in detecting SARS-CoV-2. Indeed, for all target genes, the KingFisher Flex and Maelstrom 9600 had sensitivities of 99.2% and 98.3%, respectively, with both having specificities of 100% when compared with the MagNA Pure 96 platform. The extraction performances of the three platforms were consistent regardless of the specimens (Table 3) and variant, such as SARS-CoV-2 lineage BA.1 (Table S1).

The evaluation of the technical properties of the two platforms (KingFisher Flex and Maelstrom 9600) revealed that the modified reagent had an open system, with components such as binding, washing, and an elution buffer similar to that of the original reagent. Both required only 30 min to extract nucleic acids, compared to the MagNA Pure 96 system, which required 60 min (Table 1). Under these conditions, the KingFisher Flex and Maelstrom 9600 provide an advantage when extracting DNA from a large number of clinical specimens, including NPS, saliva, and sputum.

This study had a few limitations. First, the three platforms were compared in terms of their ability to extract and analyze nucleic acids from specimens with possible SARS-CoV-2 infection, followed by mRT-qPCR analysis; other respiratory viruses and bacteria were not considered. Second, other types of respiratory samples, such as bronchoalveolar lavage and throat swabs, were not evaluated. Thus, studies are needed to determine the ability and performance of these platforms with regard to the analysis and extraction of other viruses and bacteria, as well as SARS-CoV-2 in the infected specimens not considered in the present study.

## 5. Conclusions

To the best of our knowledge, this is the first study to compare the performance of two newly launched kits (careGENE^TM^ Viral/Pathogen HiFi Nucleic Acid Isolation kit and SGRespi^TM^ Pure kit), which use open platforms such as KingFisher Flex and Maelstrom 9600, with that of the established MagNA Pure 96 platform. The KingFisher Flex and Maelstrom 9600 platforms were highly efficient in extracting SARS-CoV-2 RNA from clinical samples. Thus, these platforms can serve as highly useful alternatives to the MagNA Pure 96 system and can extract nucleic acids stably and rapidly, contributing to the quick control of pathogen transmission in the population.

## Figures and Tables

**Table 1:** Overview of the three fully automated extraction platforms used in this study.

Platform	MagNA Pure 96	KingFisher Flex	Maelstrom 9600
Platform manufacturer	Roche	Thermo Fisher Scientific	Taiwan Advanced Nanotech Inc.
Properties	Fully automated	Fully automated	Fully automated
Machine measurements (W × D × H)	136 cm × 81.5 cm × 100 cm	68 cm × 60 cm × 38 cm	87 cm × 57.5 cm × 70 cm
Extraction reagent	MagNA Pure 96 DNA and Viral NA Small Volume kit	careGENE^TM^ Viral/Pathogen HiFi Nucleic Acid Isolation kit	SGRespi^TM^ Pure kit
Reagent manufacturer	Roche	WELLS BIO	Seegene
Number of samples	96	96	96
Extraction technique	Magnetic beads	Magnetic beads	Magnetic beads
Duration of extraction	Approximately 60 min	Approximately 30 min	Approximately 30 min
Sample loading	Needed to load the sample into an empty cartridge	Needed to load the sample into a lysis buffer cartridge	Needed to load the sample into a lysis buffer cartridge
Nucleic acid collection	Nucleic acid transferred to a 96-well plate	Nucleic acid transferred to a 96-well plate	Nucleic acid transferred to a 96-well plate
Identification reader	Barcode system	Not available	Barcode system

**Table 2:** Coefficients of variation of the three platforms for the SARS-CoV-2 target.

Extraction Platform	Specimen	Analyte (CV [%] ± SD)		
		E Gene	RdRP and S Gene	N Gene
MagNA Pure 96	NPS	1.70 ± 0.49	3.28 ± 0.76	1.58 ± 0.45
	Sputum	2.80 ± 0.84	3.21 ± 0.95	2.53 ± 0.78
	Saliva	3.24 ± 1.00	3.87 ± 1.19	2.52 ± 0.83
KingFisher Flex	NPS	1.32 ± 0.36	3.81 ± 0.81	1.53 ± 0.43
	Sputum	2.14 ± 0.66	2.16 ± 0.67	1.78 ± 0.55
	Saliva	1.45 ± 0.45	1.76 ± 0.58	1.54 ± 0.52
Maelstrom 9600	NPS	1.18 ± 0.31	2.48 ± 0.53	1.70 ± 0.44
	Sputum	2.21 ± 0.66	1.92 ± 0.59	2.16 ± 0.65
	Saliva	1.10 ± 0.36	1.21 ± 0.38	1.62 ± 0.55

Data are presented as CV ± standard deviation (*n* = 120). Abbreviations: E gene, gene encoding the envelope protein of SARS-CoV-2; RdRP gene, gene encoding the RNA-dependent RNA polymerase of SARS-CoV-2; S gene, gene encoding spike protein of SARS-CoV2; N gene, gene encoding the nucleocapsid protein of SARS-CoV-2; CV, coefficient of variability; NPS, nasopharyngeal swab.

**Table 3:** Comparison of the MagNA Pure 96 with the KingFisher Flex and Maelstrom 9600 platforms.

Specimens	Target Gene	Platform	No. of Samples				Sen. (%)	Spe. (%)
			TP (+/+)	FN (+/–)	FP (–/+)	TN (–/–)		
NPS	E gene	KingFisher Flex	57	0	0	25	100	100
		Maelstrom 9600	57	0	0	25	100	100
	RdRP and S gene	KingFisher Flex	56	1	0	25	98.2	100
		Maelstrom 9600	57	0	0	25	100	100
	N gene	KingFisher Flex	57	0	0	25	100	100
		Maelstrom 9600	56	1	0	25	98.2	100
Sputum	E gene	KingFisher Flex	45	0	0	34	100	100
		Maelstrom 9600	45	0	0	34	100	100
	RdRP and S gene	KingFisher Flex	45	0	0	34	100	100
		Maelstrom 9600	45	0	0	34	100	100
	N gene	KingFisher Flex	45	0	0	34	100	100
		Maelstrom 9600	44	1	0	34	97.8	100
Saliva	E gene	KingFisher Flex	17	1	0	66	94.4	100
		Maelstrom 9600	18	0	0	66	100	100
	RdRP and S gene	KingFisher Flex	18	0	0	66	100	100
		Maelstrom 9600	18	0	0	66	100	100
	N gene	KingFisher Flex	17	1	0	66	94.4	100
		Maelstrom 9600	18	0	0	66	100	100
Total (NPS + sputum + saliva)	E gene	KingFisher Flex	119	1	0	125	99.2	100
		Maelstrom 9600	120	0	0	125	100	100
	RdRP and S gene	KingFisher Flex	119	1	0	125	99.2	100
		Maelstrom 9600	120	0	0	125	100	100
	N gene	KingFisher Flex	119	1	0	125	99.2	100
		Maelstrom 9600	118	2	0	125	98.3	100

Abbreviations: TP, true positive; FN, false negative; FP, false positive; TN, true negative; sen, sensitivity; spe, specificity; E gene, the gene encoding the envelope protein of SARS-CoV-2; RdRP gene, the gene encoding the RNA-dependent RNA polymerase of SARS-CoV-2; S gene, the gene encoding spike protein of SARS-CoV2; N gene, the gene encoding the nucleocapsid protein of SARS-CoV-2; NPS, nasopharyngeal swab.

**Table 4:** Evaluation of detection limit in the target regions.

Positive Strain	Target	Conc. (TCID_50_/mL)	E Gene		RdRp and S Gene		N Gene	
			Positive Rate (%)	LOD 95% (TCID_50_/mL)	Positive Rate (%)	LOD 95% (TCID_50_/mL)	Positive Rate (%)	LOD 95% (TCID_50_/mL)
Zeptometrix- 0810589CFHI	MagNA Pure 96	7.6 × 10^4^	100	1.74 × 10^1^	100	3.15 × 10^1^	100	1.34 × 10^1^
		7.6 × 10^3^	100		100		100	
		7.6 × 10^2^	100		100		100	
		7.6 × 10^1^	100		100		100	
		7.6 × 10^0^	62.5		31.3		75	
		7.6 × 10^−1^	0		0		0	
	KingFisher Flex	7.6 × 10^4^	100	1.34 × 10^1^	100	3.62 × 10^1^	100	9.72
		7.6 × 10^3^	100		100		100	
		7.6 × 10^2^	100		100		100	
		7.6 × 10^1^	100		100		100	
		7.6 × 10^0^	75		25		87.5	
		7.6 × 10^−1^	0		0		0	
	Maelstrom 9600	7.6 × 10^4^	100	1.98 × 10^1^	100	1.98 × 10^1^	100	1.16 × 10^1^
		7.6 × 10^3^	100		100		100	
		7.6 × 10^2^	100		100		100	
		7.6 × 10^1^	100		100		100	
		7.6 × 10^0^	56.3		56.3		81.3	
		7.6 × 10^−1^	0		0		0	
Zeptometrix- 0810590CFHI	MagNA Pure 96	1.4 × 10^3^	100	3.7 × 10^−1^	100	5.3 × 10^−1^	100	4.5 × 10^−1^
		1.4 × 10^2^	100		100		100	
		1.4 × 10^1^	100		100		100	
		1.4 × 10^0^	100		100		100	
		1.4 × 10^−1^	56.3		31.3		43.8	
		1.4 × 10^−2^	0		0		0	
	KingFisher Flex	1.4 × 10^3^	100	6.5 × 10^−1^	100	5.3 × 10^−1^	100	4.1 × 10^−1^
		1.4 × 10^2^	100		100		100	
		1.4 × 10^1^	100		100		100	
		1.4 × 10^0^	100		100		100	
		1.4 × 10^−1^	18.8		31.3		50	
		1.4 × 10^−2^	0		0		0	
	Maelstrom 9600	1.4 × 10^3^	100	3.3 × 10^−1^	100	4.5 × 10^−1^	100	8.8 × 10^−1^
		1.4 × 10^2^	100		100		100	
		1.4 × 10^1^	100		100		100	
		1.4 × 10^0^	100		100		93.8	
		1.4 × 10^−1^	62.5		43.8		68.8	
		1.4 × 10^−2^	0		0		0	

PCR reactions performed using serial ten-fold diluted virus-positive samples. The LOD 95% data were estimated using the probit regression analysis. Abbreviations: Conc., concentration; TCID_50_, median tissue culture infective dose; LOD, limit of detection.

## Data Availability

All data are available within the article.

## Supplementary Materials

The following are available online at https://www.mdpi.com/article/10.3390/life12010068/s1, Table S1: Five nasopharyngeal swab specimens used to evaluate the extraction performance of the three kits for the detection of SARS-CoV-2.

## References

[1] Hong K.H., Lee S.W., Kim T.S., Huh H.J., Lee J., Kim S.Y., Park J.S., Kim G.J., Sung H., Roh K.H. (2020). Guidelines for Laboratory Diagnosis of Coronavirus Disease 2019 (COVID-19) in Korea. Ann. Lab. Med..

[2] Gisondi P., S P.I., Bordin C., Alaibac M., Girolomoni G., Naldi L. (2020). Cutaneous manifestations of SARS-CoV-2 infection: A clinical update. J. Eur. Acad. Dermatol. Venereol..

[3] World Health Organization WHO Coronavirus (COVID-19) Dashboard. https://covid19.who.int.

[4] Zupin L., Licen S., Milani M., Clemente L., Martello L., Semeraro S., Fontana F., Ruscio M., Miani A., Crovella S. (2021). Evaluation of Residual Infectivity after SARS-CoV-2 Aerosol Transmission in a Controlled Laboratory Setting. Int. J. Environ. Res. Public Health.

[5] Munawar H.S., Khan S.I., Qadir Z., Kiani Y.S., Kouzani A.Z., Mahmud M.A.P. (2021). Insights into the Mobility Pattern of Australians during COVID-19. Sustainability.

[6] Lin E.-C., Tu H.-P., Hong C.-H. (2021). Halved Incidence of Scrub Typhus after Travel Restrictions to Confine a Surge of COVID-19 in Taiwan. Pathogens.

[7] Rajabi A., Mantzaris A.V., Mutlu E.C., Garibay O.O. (2021). Investigating Dynamics of COVID-19 Spread and Containment with Agent-Based Modeling. Appl. Sci..

[8] McFee D.R.B. (2020). COVID-19 Laboratory Testing/CDC Guidelines. Dis. Mon..

[9] Bruce E.A., Huang M.L., Perchetti G.A., Tighe S., Laaguiby P., Hoffman J.J., Gerrard D.L., Nalla A.K., Wei Y., Greninger A.L. (2020). Direct RT-qPCR detection of SARS-CoV-2 RNA from patient nasopharyngeal swabs without an RNA extraction step. PLoS Biol..

[10] Ravi N., Cortade D.L., Ng E., Wang S.X. (2020). Diagnostics for SARS-CoV-2 detection: A comprehensive review of the FDA-EUA COVID-19 testing landscape. Biosens. Bioelectron..

[11] Al-Saud H., Al-Romaih K., Bakheet R., Mahmoud L., Al-Harbi N., Alshareef I., Judia S.B., Aharbi L., Alzayed A., Jabaan A. (2020). Automated SARS-COV-2 RNA extraction from patient nasopharyngeal samples using a modified DNA extraction kit for high throughput testing. Ann. Saudi Med..

[12] Esbin M.N., Whitney O.N., Chong S., Maurer A., Darzacq X., Tjian R. (2020). Overcoming the bottleneck to widespread testing: A rapid review of nucleic acid testing approaches for COVID-19 detection. RNA.

[13] Lubke N., Senff T., Scherger S., Hauka S., Andree M., Adams O., Timm J., Walker A. (2020). Extraction-free SARS-CoV-2 detection by rapid RT-qPCR universal for all primary respiratory materials. J. Clin. Virol..

[14] Azmi I., Faizan M.I., Kumar R., Raj Yadav S., Chaudhary N., Kumar Singh D., Butola R., Ganotra A., Datt Joshi G., Deep Jhingan G. (2021). A Saliva-Based RNA Extraction-Free Workflow Integrated with Cas13a for SARS-CoV-2 Detection. Front. Cell. Infect. Microbiol..

[15] Edelmann A., Eichenlaub U., Lepek S., Kruger D.H., Hofmann J. (2013). Performance of the MagNA Pure 96 system for cytomegalovirus nucleic acid amplification testing in clinical samples. J. Clin. Microbiol..

[16] Hindiyeh M., Mor O., Pando R., Mannasse B., Kabat A., Assraf-Zarfati H., Mendelson E., Sofer D., Mandelboim M. (2019). Comparison of the new fully automated extraction platform eMAG to the MagNA PURE 96 and the well-established easyMAG for detection of common human respiratory viruses. PLoS ONE.

[17] Lau H.K., Clotilde L.M., Lin A.P., Hartman G.L., Lauzon C.R. (2013). Comparison of IMS Platforms for detecting and recovering *Escherichia coli* O157 and *Shigella flexneri* in foods. J. Lab. Autom..

[18] Arena F., Pollini S., Rossolini G.M., Margaglione M. (2021). Summary of the Available Molecular Methods for Detection of SARS-CoV-2 during the Ongoing Pandemic. Int. J. Mol. Sci..

[19] Mengelle C., Mansuy J.M., Sandres-Saune K., Barthe C., Boineau J., Izopet J. (2012). Prospective evaluation of a new automated nucleic acid extraction system using routine clinical respiratory specimens. J. Med. Virol..

[20] Morecchiato F., Coppi M., Baccani I., Maggini N., Ciccone N., Antonelli A., Rossolini G.M. (2021). Evaluation of extraction-free RT-PCR methods for faster and cheaper detection of SARS-CoV-2 using two commercial systems. Int. J. Infect. Dis..

[21] So M.K., Park S., Lee K., Kim S.K., Chung H.S., Lee M. (2021). Variant Prediction by Analyzing RdRp/S Gene Double or Low Amplification Pattern in Allplex SARS-CoV-2 Assay. Diagnostics.

[22] Lim H.J., Park J.E., Park M.Y., Baek J.H., Jung S., Sung N., Yang J.H., Lee M.W., Lee S.H., Yang Y.J. (2021). Assay System for Simultaneous Detection of SARS-CoV-2 and Other Respiratory Viruses. Diagnostics.

[23] Kubis P., Materniak M., Kuzmak J. (2013). Comparison of nested PCR and qPCR for the detection and quantitation of BoHV6 DNA. J. Virol. Methods.

[24] van Kasteren P.B., van der Veer B., van den Brink S., Wijsman L., de Jonge J., van den Brandt A., Molenkamp R., Reusken C., Meijer A. (2020). Comparison of seven commercial RT-PCR diagnostic kits for COVID-19. J. Clin. Virol..

[25] Wallis R.S. (1991). PROBIT: A computer program analysis. J. Immunol. Methods.

[26] Bland J.M., Altman D.G. (1986). Statistical methods for assessing agreement between two methods of clinical measurement. Lancet.

[27] Cheng M.P., Papenburg J., Desjardins M., Kanjilal S., Quach C., Libman M., Dittrich S., Yansouni C.P. (2020). Diagnostic Testing for Severe Acute Respiratory Syndrome-Related Coronavirus 2: A Narrative Review. Ann. Intern. Med..

[28] Yurkovetskiy L., Wang X., Pascal K.E., Tomkins-Tinch C., Nyalile T.P., Wang Y., Baum A., Diehl W.E., Dauphin A., Carbone C. (2020). Structural and Functional Analysis of the D614G SARS-CoV-2 Spike Protein Variant. Cell.

[29] Chan J.F., Yip C.C., To K.K., Tang T.H., Wong S.C., Leung K.H., Fung A.Y., Ng A.C., Zou Z., Tsoi H.W. (2020). Improved Molecular Diagnosis of COVID-19 by the Novel, Highly Sensitive and Specific COVID-19-RdRp/Hel Real-Time Reverse Transcription-PCR Assay Validated In Vitro and with Clinical Specimens. J. Clin. Microbiol..

[30] Rossmanith P., Wagner M. (2011). A novel poisson distribution-based approach for testing boundaries of real-time PCR assays for food pathogen quantification. J. Food Prot..

[31] Wee S.K., Sivalingam S.P., Yap E.P.H. (2020). Rapid Direct Nucleic Acid Amplification Test without RNA Extraction for SARS-CoV-2 Using a Portable PCR Thermocycler. Genes.

