# Supplementary material for: Evaluation of Three Automated Extraction Systems for the Detection of SARS-CoV-2 from Clinical Respiratory Specimens
Source: Life (Basel). 2022 Jan 4;12(1):68. doi: 10.3390/life12010068 (PMC8777774; doi:10.3390/life12010068)
Supplement: Supplementary file 1 [file life-12-00068-s001.zip › life-1502551-supplementary.pdf]

life

MDPI

Supplementary Materials

Supplementary Material and Method

2.1. Next-generation sequencing and data analysis

Nucleic acids were extracted from the five nasopharyngeal swab specimens using the MagNA Pure 96 platform, KingFisher Flex platform, and Maelstrom 9600 platform, simultaneously. For next-generation sequencing using the Illumina MiSeq platform (Illumina, San Diego, CA, USA), the SARS-CoV-2 FLEX panels (Paragon Genomics, Hayward, CA, USA) was used as described previously [1]. The NGS assays were analyzed using the Flomics pipeline (Flomics, Barcelona, Spain).

Table S1. Five nasopharyngeal swab specimens used to evaluate the extraction performance of the three kits for the detection of SARS-CoV-2.

Sample	*Lineage*	*MagNA Pure 96*	*KingFisher Flex*	*Maelstrom 9600*	*Comment*
A	*BA.1*	*SARS-CoV-2*	*SARS-CoV-2*	*SARS-CoV-2*	*Match*
B	*BA.1*	*SARS-CoV-2*	*SARS-CoV-2*	*SARS-CoV-2*	*Match*
C	*BA.1*	*SARS-CoV-2*	*SARS-CoV-2*	*SARS-CoV-2*	*Match*
D	*BA.1*	*SARS-CoV-2*	*SARS-CoV-2*	*SARS-CoV-2*	*Match*
E	*BA.1*	*SARS-CoV-2*	*SARS-CoV-2*	*SARS-CoV-2*	*Match*

Strain description, cited by the PANGO lineages from which the SARS-CoV-2 strain was acquired, is shown. Abbreviations: SARS-CoV-2, severe acute respiratory syndrome-related coronavirus 2.

Supplementary Reference

1. Lim, H.J.; Park, M.Y.; Jung, H.S.; Kwon, Y.; Kim, I.; Kim, D.K.; Yu, N.; Sung, N.; Lee, S.H.; Park, J.E.; Yang, Y.J. Development of an efficient Sanger sequencing-based assay for detecting SARS-CoV-2 spike mutations. PLoS One **2021**, 16**,** e0260850, doi: 10.1371/journal.pone.0260850.
